# Supplementary material for: Topological superconductivity in monolayer transition metal dichalcogenides
Source: Nat Commun. 2017 Apr 11;8:14985. doi: 10.1038/ncomms14985 (PMC5394266; doi:10.1038/ncomms14985)
Supplement: Supplementary Information — Supplementary Notes and Supplementary References [file ncomms14985-s1.pdf]

Supplementary Note 1: Why only pairing instabilities?

In this work we consider only pairing instabilities but not particle-hole instabilities, e.g. spin density waves. In the following, we explain why the pairing instabilities are expected to dominate over particle-hole instabilities in the low energy limit.

Whether instabilities in particle-hole channel or particle-particle (pairing) channel dominate depends on which of the non-interacting static susceptibilities in particle-hole channel $\Pi_{ph}(\mathbf{p})$ and particle-particle channel $\Pi_{pp}(\mathbf{p})$ diverges faster as approaching the low energy limit. These susceptibilities of electrons with spin s and low-energy dispersion $\epsilon^s(\mathbf{k})$ have the form

$$\Pi^{ss'}_{pp}(\mathbf{p}) \equiv \sum_n \int \frac{d^2k}{4\pi^2} G^s(i\omega_n, \mathbf{k}) G^{s'}(-i\omega_n, -\mathbf{k}+\mathbf{p}) = \int \frac{d^2k}{(2\pi)^2} \frac{1 - f(\epsilon^s_{-\mathbf{k}+\mathbf{p}}) - f(\epsilon^{s'}_{\mathbf{k}})}{\epsilon^s(-\mathbf{k}+\mathbf{p}) + \epsilon^{s'}(\mathbf{k})} \quad (1)$$

and

$$\Pi^{ss'}_{ph}(\mathbf{p}) \equiv -\sum_n \int \frac{d^2k}{4\pi^2} G^s(i\omega_n, \mathbf{k}) G^{s'}(i\omega_n, \mathbf{k}+\mathbf{p}) = -\int \frac{d^2k}{(2\pi)^2} \frac{f(\epsilon^s_{\mathbf{k}+\mathbf{p}}) - f(\epsilon^{s'}_{\mathbf{k}})}{\epsilon^s(\mathbf{k}+\mathbf{p}) - \epsilon^{s'}(\mathbf{k})}, \quad (2)$$

where spin $s, s' = \uparrow / \downarrow$, ω_n is the fermionic Matsubara frequency, $\mathbf{k}$ and $\mathbf{p}$ are momenta, $G^s(i\omega_n, \mathbf{k}) = \frac{1}{i\omega_n - \epsilon^s(\mathbf{k})}$ is the non-interacting Green's function, and $f(\epsilon^s_{\mathbf{k}})$ is the Fermi function at temerature T.

In general, $\Pi^{ss'}_{pp}(\mathbf{p})$ always diverges logarithmically at total-momentum $\mathbf{p} = 0$ despite the low-energy dispersion $\epsilon^s_{\mathbf{k}}$, which indicates pair-momentum 0 superconductivity if dominates. On the other hand, $\Pi^{ss'}_{ph}(\mathbf{p})$ typically diverges at momentum-transfer $\mathbf{p} = 0$ when the density of states diverges, i.e. near the van Hover singularity, or at some finite momentum-transfer $\mathbf{p} = \mathbf{Q}$ when the Fermi surface is nested in the particle-hole channel at $\mathbf{Q}$. The former and latter indicate instabilities such as ferromagnetism and density-waves respectively when they each dominates. In a two-pocket system, this requires a hole and an electron pocket to have the same low-energy dispersion (but opposite in energy). In the case where susceptibilities in the two channels diverge equally fast, one needs to further compare their corresponding driving interactions to determine the dominant instability.

In the current lightly p-doped monolayer TMD case, note that both pockets are hole pockets though they have the same low-energy dispersion $\epsilon^{\uparrow}(\mathbf{k}) = -\frac{(\mathbf{k}-\mathbf{K})^2}{2m}$ and $\epsilon^{\downarrow}(\mathbf{k}) = -\frac{(\mathbf{k}+\mathbf{K})^2}{2m}$ with respect to their own valley centers $\mathbf{K}$ and $-\mathbf{K}$ upon low-doping. Thus, the

Fermi surface is in fact poorly nested at $2\mathbf{K}$ in the particle-hole channel. To be precise, since $\epsilon^{\downarrow}(\mathbf{p}) = \epsilon^{\uparrow}(\mathbf{p}+2\mathbf{K})$, the particle-hole susceptibility has the relation

$$\Pi^{s\bar{s}}_{ph}(2\mathbf{K}+\mathbf{p}) = -\int \frac{d^2k}{4\pi^2} \frac{f^{s}_{\mathbf{k}+2\mathbf{K}+\mathbf{p}} - f^{\bar{s}}_{\mathbf{p}}}{\epsilon^{s}(\mathbf{k}+2\mathbf{K}+\mathbf{p}) - \epsilon^{\bar{s}}(\mathbf{k})} = -\int \frac{d^2k}{4\pi^2} \frac{f^{\bar{s}}_{\mathbf{k}+\mathbf{p}} - f^{\bar{s}}_{\mathbf{k}}}{\epsilon^{\bar{s}}(\mathbf{k}+\mathbf{p}) - \epsilon^{\bar{s}}(\mathbf{k})} = \Pi^{\bar{s}\bar{s}}_{ph}(\mathbf{p}) \tag{3}$$

for $s=\uparrow$ and $\bar{s}=-s$. Thus,

$$\Pi^{s\bar{s}}_{ph}(\pm 2\mathbf{K}) = \Pi^{ss}_{ph}(0) \sim \nu_0 \tag{4}$$

is not diverging in the low energy limit as long as the density of states on the Fermi surface ν_0 is finite. Therefore, we do not consider particle-hole susceptibilities in this work.

On the other hand, since the Fermi surface is perfectly nested at $2\mathbf{K}$ in the particle-particle channel, the particle-particle susceptibility

$$\Pi^{s\bar{s}}_{pp}(0) = \Pi^{ss}_{pp}(\pm 2\mathbf{K}) \sim \nu_0 \mathrm{Log}(\frac{\Lambda}{E}) \tag{5}$$

diverges logarithmically as approaching the low-energy limit $E \to 0$ with Λ being the UV cutoff scale. Note that the Cooper logarithmic divergence does not occur only at the usual $\mathbf{p}=0$, but also at $\mathbf{p}=2\mathbf{K}$. This indicates that the superconductivity with pair momentum 0 (spatially uniform) and $2\mathbf{K}$ (spatially modulated at $2\mathbf{K}$) could be equally dominant in the low energy. To determine which is truly more dominant, we need to study their pairing interactions using the RG analysis in the following section.

Supplementary Note 2: Inter- and intra-pocket effecitve interactions

We will calculate the inter- and intra-valley effective interactions $g^{(0)}_{\mathrm{inter}}(\mathbf{q},\mathbf{q}') \equiv g^{(0)}_{\tau,\bar{\tau}}(\mathbf{q},\mathbf{q}')$ and $g^{(0)}_{\mathrm{intra}}(\mathbf{q},\mathbf{q}') \equiv g^{(0)}_{\tau,\tau}(\mathbf{q},\mathbf{q}')$ at energy Λ_0 in terms of the incoming and outgoing momenta $\mathbf{q}$ and $\mathbf{q}'$ order by order in U until we obtain attraction in one of them in certain partial-wave channel $\tilde{l}$. Before we start, notice that by omitting the valley index, which is inter-locked with the spin index τ for the low-energy fermions, the inter- and intra-valley interactions in the spin-valley locked two-pocket picture are just the opposite- and equal-spin interactions in a spin-degenerate single-pocket picture. Fortunately, Ref. 1 has already studied the pairing problem in a spin-degenerate single-pocket system under repulsive Hubbard interaction described by Eq. (1) and Eq. (2). Thus, we expect the same result as Ref.

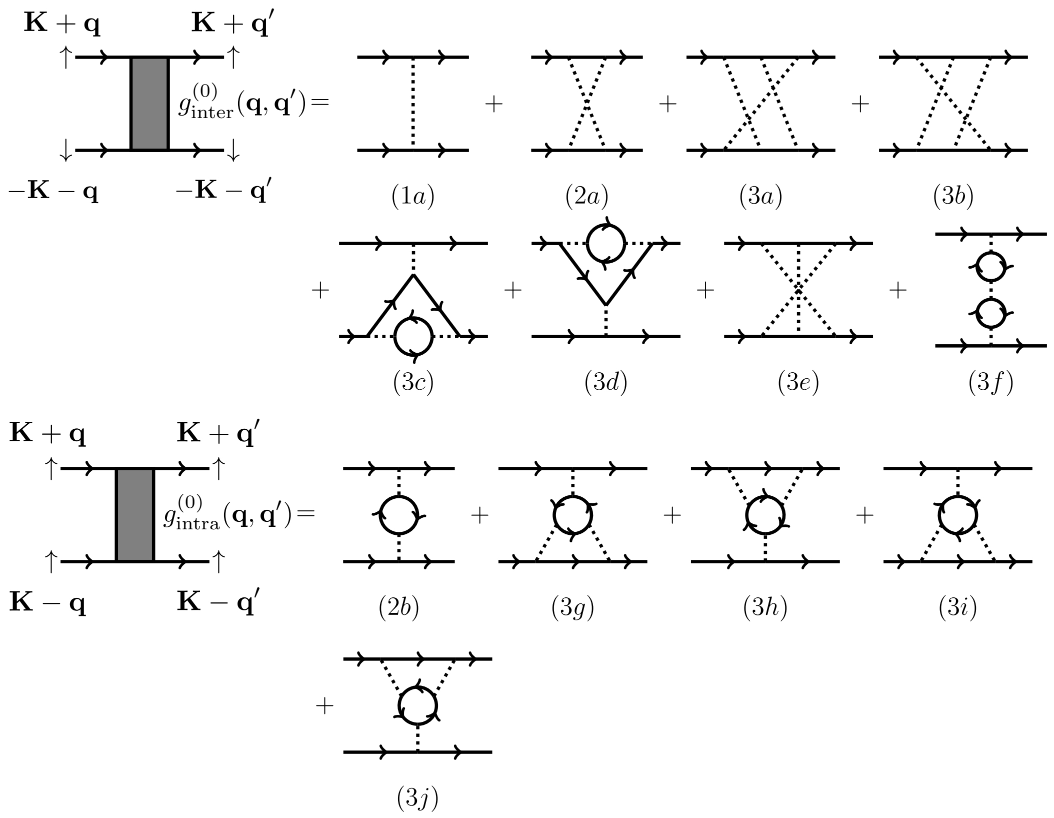

Supplementary Fig. 1: Feymann diagrams for the contributions up to two-loop order to the inter- and intra-pocket effective interactions $g^{(0)}_{\rm inter/intra}(\mathbf{q},\mathbf{q}')$ at the intermediate energy scale $E=\Lambda_0$. The solid and dotted lines represent fermions and repulsive Hubbard interaction U respectively.

1, i.e. the largest attraction occuring in the angular-momentum-one channel, but with a different physical meaning when mapping back to the spin-valley locked two-pocket picture. To make the mapping between the two pictures explicit, we will follow Ref. 1 to calculate $g^{(0)}_{\rm inter}(\mathbf{q},\mathbf{q}')$ and $g^{(0)}_{\rm intra}(\mathbf{q},\mathbf{q}')$ in the two-pocket picture and denote the spin s and valley $\pm\mathbf{K}$ separately.

2.1 Tree level

At the tree level, the on-site repulsion $U>0$ only contribute to the inter-pocket interaction because U acts between only electrons with opposite spins due to Pauli exclusive principle. Thus,

$$g^{(0),1}_{\rm inter}(\mathbf{q},\mathbf{q}')=U \tag{6}$$

with the superscript 1 denoting the tree-level contribution [see Supplementary Fig. 1(1a)]. This bare repulsion contributes to only the $\tilde{l} = 0$ component of $g^{(0)}_{\text{inter}}$ because U is independent of the incoming and outgoing momenta $\mathbf{q}$ and $\mathbf{q}'$. Since this is a perturbative analysis, the inter-pocket $\tilde{l} = 0$ pairing is suppressed regardless what the higher order contributions to $\tilde{l} = 0$ channel are. To have any finite contribution to aisotropic channels ($\tilde{l} \neq 0$) requires momentum-dependence from loop corrections.

2.2 Second order

The U^2 (one-loop) order contributions to inter- and intra-pocket interactions are

$$g^{(0),2}_{\text{inter}}(\mathbf{q}, \mathbf{q}') = U^2 \Pi^{s\bar{s}}_{ph}(\pm\mathbf{Q} + \mathbf{q} + \mathbf{q}'), \tag{7}$$

and

$$g^{(0),2}_{\text{intra}}(\mathbf{q}, \mathbf{q}') = -U^2 \Pi^{ss}_{ph}(\mathbf{q} - \mathbf{q}') \tag{8}$$

respectively for $s = \uparrow / \downarrow$, where the superscript 2 denotes corrections from the second order [see Supplementary Fig. 1(2a) and (2b)]. The particle-hole susceptibilies defined in Supplementary Eq. (2) can be calculated as

$$\Pi^{ss}_{ph}(\mathbf{p}) = \frac{m}{2\pi^2} \int_{-\pi/2}^{\pi/2} d\phi \int_{k_2}^{k_1} dk \frac{k}{kp\cos\phi} = \frac{m}{2\pi} = \Pi^{\bar{s}s}_{ph}(\mathbf{p}) \tag{9}$$

where $k_{1/2} \equiv \pm\frac{p}{2}\cos\phi + \frac{\sqrt{4q_F^2 - p^2\sin^2\phi}}{2}$. Thus, the one-loop corrections are still momentum-independent and contribute to only the $\tilde{l} = 0$ channel. This is a consequence of isotropic parabolic dispersion in 2D.[1,2] Note that though $g^{(0),2}_{\text{intra}}$ seems to imply $\tilde{l} = 0$ intra-pocket pairing, pairings in even $\tilde{l}$ channels are not allowed since these are equal-spin pairs. Thus, if either the inter- or intra-pocket pairing were to occur at all, the effective attraction has to come from at least two-loop order.

2.3 Third order

The U^3 (two-loop) contribution of short-range repulsion for a spin-degenerate rotational-invariant 2D system with a single pocket and parabolic dispersion has been proven to facilitate p-wave (angular momentum 1) pairing.[1] Since both pockets in p-doped TMDs have the

same low-energy effective dispersion which is parabolic upon light doping, we can map this spin-valley locked two-pocket system to the spin-degenerate single-pocket system studied in Ref.1 by bringing the pocket centers K and K' both to $\mathbf{k} = 0$. Thus, we expect to obtain the largest attractions in the partial-wave channel $\tilde{l} = 1$ as well, but the partial-wave channels here are with respect to K and K' instead of Γ. This indicates degenerate inter- and intra-pocket pairings with $\tilde{l} = 1$ after we map back to the two-pocket system. In the following, we will show the calculations of $g^{(0)}_{\text{intra/inter}}$ following Ref. 1 to comfirm our expectation.

From the corresponding diagrammatic expressions shown in Supplementary Fig. 1(3a)-(3j), we can see that the two-loop contributions can be divided into two groups: the ones with one particle-particle and one particle-hole bubble (diagram 3a, 3b, 3g and 3h), and the ones with two particle-hole bubbles (diagram 3c, 3d, 3e, 3f, 3i, and 3j). We first calculate the former contributions to intra-pocket interaction, i.e. diagram (3g) and (3h). In the static limit,

$$
\begin{aligned}
g^{pp}_{\text{intra}}(\mathbf{q},\mathbf{q}') &= g_{3g}(\mathbf{q},\mathbf{q}') + g_{3h}(\mathbf{q},\mathbf{q}') \\
&= -U^3 \sum_{n,\tilde{n}} \int \frac{d^2 l}{4\pi^2} \int \frac{d^2\tilde{l}}{4\pi^2} G^{\uparrow}(i\omega_{\tilde{n}}, \tilde{\mathbf{l}}) G^{\downarrow}(-i\omega_{\tilde{n}}, -\tilde{\mathbf{l}} + \mathbf{t}_+) G^{\downarrow}(i\omega_n, \mathbf{l} + \frac{\mathbf{p}}{2}) G^{\downarrow}(i\omega_n, \mathbf{l} - \frac{\mathbf{p}}{2}) \\
&+ (\mathbf{t}_+ \to \mathbf{t}_-)
\end{aligned} \tag{10}
$$

where $\omega_{n(\tilde{n})}$ is the fermionic Matsubara frequency, $G^s(i\omega_n, \mathbf{l}) = \frac{1}{i\omega_n - \epsilon^s(\mathbf{l})}$ is the non-interacting Green's function, $\mathbf{t}_\pm \equiv \mathbf{l} \pm \frac{\mathbf{q}+\mathbf{q}'}{2}$, $\mathbf{p} \equiv \mathbf{q}' - \mathbf{q}$, and $\mathbf{q}$ and $\mathbf{q}'$ are the external incoming and outgoing momenta relative to the valley center $\mathbf{K}$. As the electrons have energy $E = \Lambda_0 \gtrsim 0$, $|\mathbf{q}| = |\mathbf{q}'| \sim q_F$ with q_F being the Fermi momentum of a single pocket. The over-all minus sign results from the closed fermion loop. The particle-particle loop integral can be calculated as

$$
\begin{aligned}
&\sum_{\tilde{n}} \int \frac{d^2\tilde{l}}{4\pi^2} G^{\uparrow}(i\omega_{\tilde{n}}, \tilde{\mathbf{l}}) G^{\downarrow}(-i\omega_{\tilde{n}}, -\tilde{\mathbf{l}} + \mathbf{t}_+) = \int \frac{d^2\tilde{l}}{4\pi^2} \frac{1 - f(\epsilon^{\uparrow}_{\tilde{\mathbf{l}}}) - f(\epsilon^{\downarrow}_{-\tilde{\mathbf{l}}+\mathbf{t}_+})}{\epsilon^{\uparrow}_{\tilde{\mathbf{l}}} + \epsilon^{\downarrow}_{-\tilde{\mathbf{l}}+\mathbf{t}_+}} = \int \frac{d^2\tilde{l}}{4\pi^2} \frac{1 - f(\epsilon^{\uparrow}_{\tilde{\mathbf{l}}}) - f(\epsilon^{\uparrow}_{\tilde{\mathbf{l}}-\mathbf{t}_+})}{\epsilon^{\uparrow}_{\tilde{\mathbf{l}}} + \epsilon^{\uparrow}_{\tilde{\mathbf{l}}-\mathbf{t}_+}} \\
&= -2m \int \frac{d^2\tilde{l}}{4\pi^2} \frac{1 - f(\epsilon^{\uparrow}_{\tilde{\mathbf{l}}+\mathbf{K}+\frac{\mathbf{t}_+}{2}}) - f(\epsilon^{\uparrow}_{\tilde{\mathbf{l}}+\mathbf{K}-\frac{\mathbf{t}_+}{2}})}{(\tilde{\mathbf{l}} + \frac{\mathbf{t}_+}{2})^2 + (\tilde{\mathbf{l}} - \frac{\mathbf{t}_+}{2})^2 - 2q_F^2} = -\frac{2m}{4\pi^2} \int_{-\pi/2}^{\pi/2} d\tilde{\phi} \Big(\int_0^{\tilde{l}_2} - \int_{\tilde{l}_1}^{r_0^{-1}} \Big) d\tilde{l} \frac{\tilde{l}}{\tilde{l}^2 + \frac{t_+^2}{4} - q_F^2} \\
&\sim -\frac{m}{2\pi} \Big(\ln\Big[\frac{t_+^2}{q_F^2}\Big] + c_0\Big)
\end{aligned} \tag{11}
$$

assuming $t_\pm \ll 2q_F$, which is the regime where the main momentum-dependence comes from.[1] Here, $\tilde{\phi}$ is the angle between the loop momentum $\tilde{\mathbf{l}}$ and $\mathbf{t}_+$, r_0^{-1} the UV cutoff for

momentum integral, $\tilde{l}_{1/2} \equiv \pm\frac{t_+ \cos\tilde{\phi}}{2} + \frac{t_+}{2}\sqrt{\frac{4q_F^2}{t_+^2} - \sin^2\tilde{\phi}}$, and c_0 contains terms independent of $\mathbf{t}_\pm$. We will drop c_0 in the following since our purpose is to obtain the momentum-dependent part. Plugging Supplementary Eq. (11) back to Supplementary Eq. (10), we obtain the second loop integral

$$
\begin{aligned}
g^{pp}_{\text{intra}}(\mathbf{q},\mathbf{q}') &= \frac{+mU^3}{2\pi}\sum_n \int \frac{d^2l}{4\pi^2}\ln[\frac{t_+^2 t_-^2}{q_F^4}]G^{\downarrow}(i\omega_n, \mathbf{l}+\frac{\mathbf{p}}{2})G^{\downarrow}(i\omega_n, \mathbf{l}-\frac{\mathbf{p}}{2}) \\
&= \frac{-2m^2U^3}{2\pi}\int \frac{d^2l}{4\pi^2}\ln[\frac{t_+^2 t_-^2}{q_F^4}]\frac{f_{\mathbf{l}+\frac{\mathbf{p}}{2}} - f_{\mathbf{l}-\frac{\mathbf{p}}{2}}}{(\mathbf{l}+\frac{\mathbf{p}}{2})^2 - (\mathbf{l}-\frac{\mathbf{p}}{2})^2} \\
&\sim \frac{-m^2U^3}{4\pi^3}\int_0^{\pi/2}\frac{d\phi}{\cos\phi}\int_{\bar{l_2}}^{\bar{l_1}} d\bar{l}\ln[(\bar{l}^2-\epsilon^2)^2 + 4\epsilon^2\bar{l}^2\cos^2\phi]
\end{aligned} \tag{12}
$$

where ϕ is the angle between $\mathbf{p}$ and $\mathbf{l}$, $\epsilon^2 \equiv \frac{4q_F^2 - p^2}{p^2}$, $\bar{l} \equiv \frac{2l}{p}$, and $\bar{l}_{1/2} \equiv \pm\cos\phi + \sqrt{\epsilon^2 + \cos^2\phi}$. Here, $\epsilon \ll 1$ is a small parameter as we assumed $t_+/q_F \ll 1$ in the first loop, which corresponds to the regime where the external momenta satisfy $p \sim 2q_F$ and the loop momentum $l/q_F \sim \bar{l} \ll 1$. Notice that the integral is dominated by the regime of ϕ where $\cos\phi = O(\epsilon)$ is another small parameter besides ϵ. Since we are interested in the portion of scattering amplitude which depends on the external momenta, we will calculate $g^{pp}_{\text{intra}}(\mathbf{q},\mathbf{q}') - g^{pp}_{\text{intra}}(p = 2q_F)$ up to the leading order in the small parameters ϵ and $\cos\phi$. By keeping the small parameters in the upper and lower limits $\bar{l}_{1/2}$ while dropping those in the slowly varying logarithmic integrand, we obtain

$$
g^{pp}_{\text{intra}}(\mathbf{q},\mathbf{q}') - g^{pp}_{\text{intra}}(p = 2q_F) \sim -\frac{m^2U^3}{4\pi}\frac{\sqrt{4q_F^2 - p^2}}{2q_F} \tag{13}
$$

for the regime of external momenta satisfying $\epsilon \ll 1$.

The two-loop contributions to intra-pocket interaction involving only particle-hole bubbles, i.e. diagram (3i) and (3j), can be calculated in a similar way. In the same regime where the external momenta satisfy $\epsilon \ll 1$, we obtain

$$
\begin{aligned}
g^{ph}_{\text{intra}}(\mathbf{q},\mathbf{q}') &= g_{3i}(\mathbf{q},\mathbf{q}') + g_{3j}(\mathbf{q},\mathbf{q}') \\
&\propto -\frac{U^3m^2}{64\pi^3}(1 - \frac{p^2}{4q_F^2})\log[1 - \frac{p^2}{4q_F^2}],
\end{aligned} \tag{14}
$$

where the minus sign is due to the closed fermion loop.

We then turn to the inter-pocket interaction. Among all the third-order contributions to $g^{(0)}_{\text{inter}}$, diagram (3e) and (3f) in Supplementary Fig. 1 are both just the product of two

second-order corrections and do not contribute to momentum-dependence. Thus, we will focus only on diagram (3a)$\sim$ (3d). Note that similar to the case of intra-pocket interaction, diagram (3a) and (3b) involve vertex corrections from one particle-particle and one particle-hole bubble just like (3g) and (3h), while diagram (3c) and (3d) involve corrections from two particle-hole bubbles just like (3i) and (3j). Thus, diagram (3a) and (3b) have similar amplitudes as diagram (3c) and (3d) except the momentum-transfer in the particle-hole bubble and the absence of closed fermion loop:

$$g^{pp}_{\text{inter}}(\mathbf{q},\mathbf{q}') - g^{pp}_{\text{inter}}(p=2q_F) \sim \frac{m^2U^3}{4\pi}\frac{\sqrt{4q_F^2-p'^2}}{2q_F}, \tag{15}$$

where $\mathbf{p}' \equiv \mathbf{q}' + \mathbf{q}$. On the other hand, diagram (3c) and (3d) have the same amplitudes as diagram (3i) and (3j) such that

$$g^{ph}_{\text{inter}}(\mathbf{q},\mathbf{q}') = g_{3c}(\mathbf{q},\mathbf{q}') + g_{3d}(\mathbf{q},\mathbf{q}') \propto -\frac{U^3m^2}{64\pi^3}(1-\frac{p^2}{4q_F^2})\log[1-\frac{p^2}{4q_F^2}] \tag{16}$$

in the regime where ϵ is small. After collecting all the contributions, the U^3 corrections to the effective inter- and intra-pocket interactions at $E=\Lambda_0$ read

$$\begin{aligned} g^{(0),3}_{\text{inter}}(\mathbf{q},\mathbf{q}') &= g^{pp}_{\text{inter}}(\mathbf{q},\mathbf{q}') + g^{ph}_{\text{inter}}(\mathbf{q},\mathbf{q}') \\ &\sim \frac{m^2U^3}{2\pi^3}\frac{\sqrt{4q_F^2-p'^2}}{2q_F} - \frac{U^3m^2}{64\pi^3}(1-\frac{p^2}{4q_F^2})\log[1-\frac{p^2}{4q_F^2}] \end{aligned} \tag{17}$$

and

$$\begin{aligned} g^{(0),3}_{\text{intra}}(\mathbf{q},\mathbf{q}') &= g^{pp}_{\text{intra}}(\mathbf{q},\mathbf{q}') + g^{ph}_{\text{intra}}(\mathbf{q},\mathbf{q}') \\ &\sim -\frac{m^2U^3}{2\pi^3}\frac{\sqrt{4q_F^2-p^2}}{2q_F} - \frac{U^3m^2}{64\pi^3}(1-\frac{p^2}{4q_F^2})\log[1-\frac{p^2}{4q_F^2}]. \end{aligned} \tag{18}$$

In summary, we have derived the effective inter- and intra-pocket interactions at $E=\Lambda_0$ from the bare repulsion $U>0$ up to two-loop order:

$$\begin{aligned} g^{(0)}_{\text{inter}}(\mathbf{q},\mathbf{q}') &= g^{(0),1}_{\text{inter}}(\mathbf{q},\mathbf{q}') + g^{(0),2}_{\text{inter}}(\mathbf{q},\mathbf{q}') + g^{(0),3}_{\text{inter}}(\mathbf{q},\mathbf{q}') \\ &\sim C + \frac{m^2U^3}{2\pi^3}\frac{\sqrt{4q_F^2-p'^2}}{2q_F} - \frac{U^3m^2}{64\pi^3}(1-\frac{p^2}{4q_F^2})\log[1-\frac{p^2}{4q_F^2}], \end{aligned} \tag{19}$$

and

$$\begin{aligned} g^{(0)}_{\text{intra}}(\mathbf{q},\mathbf{q}') &= g^{(0),1}_{\text{intra}}(\mathbf{q},\mathbf{q}') + g^{(0),2}_{\text{intra}}(\mathbf{q},\mathbf{q}') + g^{(0),3}_{\text{intra}}(\mathbf{q},\mathbf{q}') \\ &\sim C' - \frac{m^2U^3}{2\pi^3}\frac{\sqrt{4q_F^2-p^2}}{2q_F} - \frac{U^3m^2}{64\pi^3}(1-\frac{p^2}{4q_F^2})\log[1-\frac{p^2}{4q_F^2}], \end{aligned} \tag{20}$$

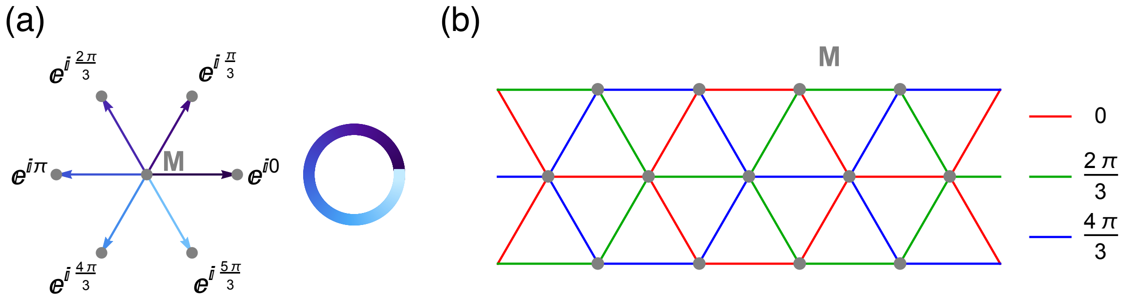

Supplementary Fig. 2: The phase of the intra-pocket pairing wave function in real space: (a) shows the phase $e^{i\theta_{\mathbf{d}}}$ in Supplementary Eq. (21) which accounts for the chiral $\tilde{l} = 1$ phase-winding within a pocket. The arrows represent $\mathbf{d}$ for the nearest-neightboring transition metal ions M (the grey dots). (b) shows the spatially modulated phase $e^{i2\mathbf{K}\cdot\mathbf{r}}$ due to the finite pair-momentum $2\mathbf{K}$ for a spin-up pair. We consider only pairing between electrons from nearest-neightboring sites. Thus, the phase of the pairing wave function is defined on each bond. The colors on the bonds represent different values of $2\mathbf{K}\cdot\mathbf{r}$.

where $C > 0$ and $C' < 0$ are momentum-independent constants.

Supplementary Note 3: The real-space profile of the phase of the intra-pocket pairing wavefunction

Since the intra-pocket pairs are spinless and the intra-pocket interaction is attractive in the $\tilde{l} = 1$ channel, the intra-pocket pairing wavefunction on the spin-up pocket is expected to be $\Delta_{\mathbf{q}}^{\uparrow\uparrow} = \langle \psi_{\mathbf{K}+\mathbf{q},\uparrow}\psi_{\mathbf{K}-\mathbf{q},\uparrow}\rangle \propto q_x \pm iq_y$ in terms of separate spin and valley indices. The p-wave pairing is expected to be chiral to avoid nodes due to energetics. The pairing wavefunction in real space can then be obtained by doing the following Fourier transform:

$$\langle \psi_{\mathbf{r}+\frac{\mathbf{d}}{2},\uparrow}\psi_{\mathbf{r}-\frac{\mathbf{d}}{2},\uparrow}\rangle = \sum_{\mathbf{q}} \langle \psi_{\mathbf{K}+\mathbf{q},\uparrow}\psi_{\mathbf{K}-\mathbf{q},\uparrow}\rangle e^{i2\mathbf{K}\cdot\mathbf{r}} e^{i\mathbf{q}\cdot\mathbf{d}} = e^{i2\mathbf{K}\cdot\mathbf{r}} \sum_{\theta_{\mathbf{q}}} q_F e^{\pm i\theta_{\mathbf{q}}} e^{iqd\cos(\theta_{\mathbf{q}}-\theta_{\mathbf{d}})} \propto e^{i2\mathbf{K}\cdot\mathbf{r}} e^{i\theta_{\mathbf{d}}} \tag{21}$$

where $\mathbf{r}$ and $\mathbf{d} = d(\cos\theta_{\mathbf{d}}, \sin\theta_{\mathbf{d}})$ are the center-of-mass and relative positions of the pair repectively, the relative momentum $\mathbf{q} = q_F(\cos\theta_{\mathbf{q}}, \sin\theta_{\mathbf{q}})$ is confined on the circular pocket centered at $\mathbf{K}$ with q_F being the Fermi momentum, and $\theta_{\mathbf{d}}(\theta_{\mathbf{q}})$ is the angle between $\mathbf{d}(\mathbf{q})$ and $\mathbf{K}$. While the phase winding from $e^{i\theta_{\mathbf{d}}}$ [see Supplementary Fig. 2(a)] accounts for the $q_x + iq_y$ pairing symmetry on a pocket, the spatial modulation in phase from $e^{i2\mathbf{K}\cdot\mathbf{r}}$ [see Supplementary Fig. 2(b)] is a consequence of the finite pair-momentum $2\mathbf{K}$.

Supplementary References

[1] Chubukov, A. V. Kohn-luttinger effect and the instability of a two-dimensional repulsive fermi liquid at $T = 0$. *Phys. Rev. B* **48**, 1097–1104 (1993).

[2] Raghu, S., Kivelson, S. A. & Scalapino, D. J. Superconductivity in the repulsive hubbard model: An asymptotically exact weak-coupling solution. *Phys. Rev. B* **81**, 224505 (2010).
